# Closed-loop vagus nerve stimulation for heart rate control evaluated in the Langendorff-perfused rabbit heart

**DOI:** 10.1038/s41598-022-23407-2

**Published:** 2022-11-05

**Authors:** Max Haberbusch, Bettina Kronsteiner, Anne-Margarethe Kramer, Attila Kiss, Bruno K. Podesser, Francesco Moscato

**Affiliations:** 1 Center for Medical Physics and Biomedical Engineering, Medical University of Vienna, Vienna, Austria; 2 Ludwig Boltzmann Institute for Cardiovascular Research, Vienna, Austria; 3 Center for Biomedical Research, Medical University of Vienna, Vienna, Austria; 4 Ludwig Boltzmann Cluster for Tissue Regeneration, Vienna, Austria

**Keywords:** Biomedical engineering, Computational models, Cardiac device therapy

## Abstract

Persistent sinus tachycardia substantially increases the risk of cardiac death. Vagus nerve stimulation (VNS) is known to reduce the heart rate, and hence may be a non-pharmacological alternative for the management of persistent sinus tachycardia. To precisely regulate the heart rate using VNS, closed-loop control strategies are needed. Therefore, in this work, we developed two closed-loop VNS strategies using an in-silico model of the cardiovascular system. Both strategies employ a proportional-integral controller that operates on the current amplitude. While one control strategy continuously delivers stimulation pulses to the vagus nerve, the other applies bursts of stimuli in synchronization with the cardiac cycle. Both were evaluated in Langendorff-perfused rabbit hearts (n = 6) with intact vagal innervation. The controller performance was quantified by rise time (T_r_), steady-state error (SSE), and percentual overshoot amplitude (%OS). In the ex-vivo setting, the cardiac-synchronized variant resulted in T_r_ = 10.7 ± 4.5 s, SSE = 12.7 ± 9.9 bpm and %OS = 5.1 ± 3.6% while continuous stimulation led to T_r_ = 10.2 ± 5.6 s, SSE = 10 ± 6.7 bpm and %OS = 3.2 ± 1.9%. Overall, both strategies produced a satisfying and reproducible performance, highlighting their potential use in persistent sinus tachycardia.

## Introduction

Cardiovascular diseases are still the major cause of death worldwide. One condition with serious implications for patient health is persistent sinus tachycardia. Persistent sinus tachycardia is the chronic elevation of the resting heart rate which leads not only to reduced patient quality of life but also to secondary diseases such as cardiomyopathy^1,2^.

Treatment of persistent sinus tachycardia can improve left ventricular function^3^ and prevent the development of secondary diseases. Typically, the heart rate can be reduced to a physiological level by pharmacological intervention, e.g., by prescription of beta blockers^4^ or antiarrhythmic drugs^3,5^. However, pharmacological therapies are associated with undesired side effects like tiredness, dizziness, or hypotension. Moreover, patients taking beta-blockers may also have an impaired heart rate response to physical activity. Finally, there is a number of patients that are not eligible for pharmacological therapy due to contraindications, raising the need for alternative treatment options.

Vagus nerve stimulation (VNS) is already a well-established therapy for diseases such as refractory epilepsy^6–10^ or treatment-resistant depression^11–14^. More recently, VNS has also been getting into focus of cardiovascular applications such as the treatment of heart failure^15–17^. Multiple studies have shown that open-loop vagus nerve stimulation (VNS) can also effectively reduce the heart rate in animals and humans^18–26^ and hence may pose an alternative therapy for the treatment of persistent sinus tachycardia. However, to maintain the heart rate at the desired level and to improve the therapeutic efficacy of VNS, closed-loop control strategies are needed^27–29^.

The goal of this study was to develop and evaluate closed-loop control strategies for the regulation of the heart to manage persistent tachycardia by stimulating proximal to the superior cardiac branch of the vagus nerve. The controller was developed using an in-silico model of the cardiovascular system and its autonomic control. A virtual study population was generated to assess and optimize the control strategy performance in the presence of interindividual variabilities in parameters associated with the cardiovascular system and autonomic cardiovascular control. Finally, the control strategy was implemented using hardware-in-the-loop tools and evaluated based on standard controller performance metrics in a series of experiments with Langendorff-perfused isolated rabbit hearts with intact vagal innervation.

A preliminary version of this work has been reported^30^.

## Methods

This chapter is structured into five parts. First, a brief outline of the employed in-silico model is given. This part is followed by an overview of the closed-loop control strategy development. Then, we describe the assessment and optimization of the control strategy based on a virtual study population. Finally, we depict the ex-vivo experiments using the Langendorff-perfused rabbit heart setup to evaluate the control strategy performance.

### Outline of the in-silico model

For the development of the control strategy, we used an in-silico model of the acute cardiac effects of vagus nerve stimulation that has been developed previously by our group^31,32^. In brief, the model comprises a lumped-parameter representation of the cardiovascular system with its systemic and pulmonary circulation, realized as modified Windkessel models, and the heart chambers modeled as non-linear time-varying elastances. It integrates autonomic cardiovascular control pathways including the arterial baroreflex and pulmonary stretch reflex. The acute cardiac effects of vagus nerve stimulation were modeled on the level of the electrode-nerve interface by a multi-axon multi-compartment Chiu-Ritchie-Rogart-Stagg model^33^ and at the level of vagal cardiac neuroeffector junctions by a model of acetylcholine dynamics^34^.

### Closed-loop control strategy development

**Figure 1:**
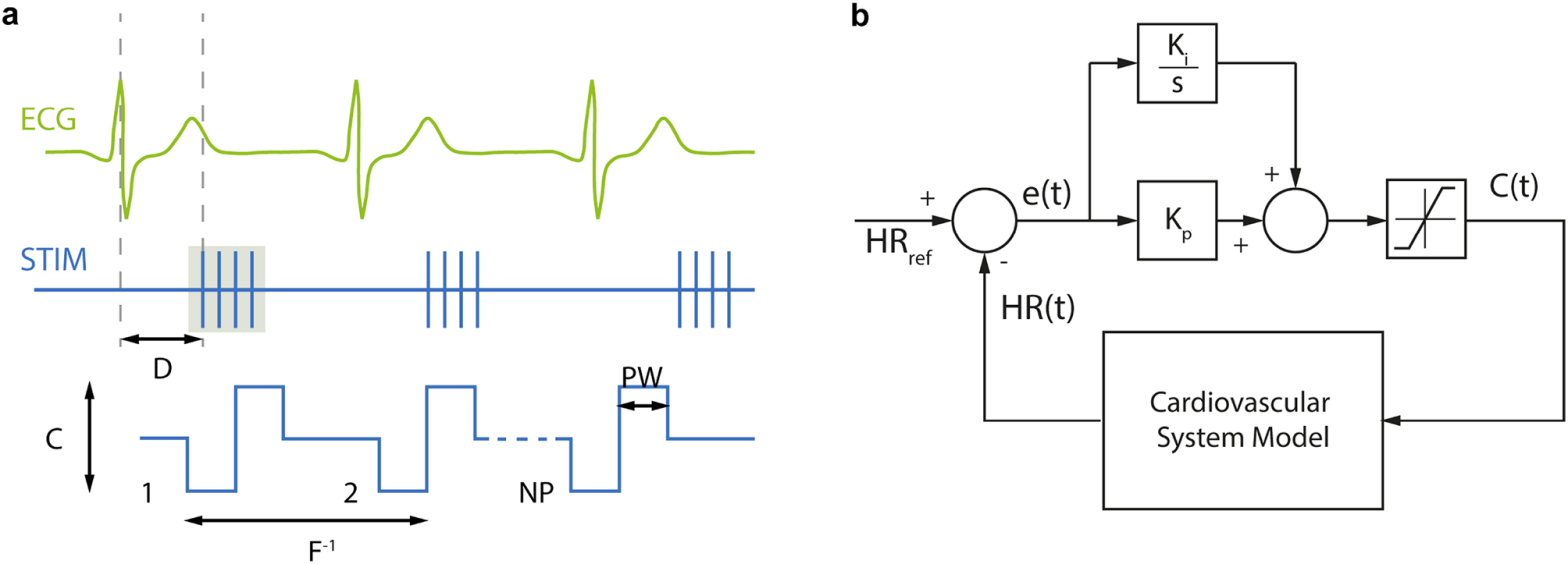
Overview of the stimulation paradigm and the control strategy implementation. (**a**) Cardiac-synchronized stimulation, as is defined by its five main parameters: current amplitude, C, pulse width, PW, frequency, F, number of pulses per burst, NP, and stimulation onset delay, D, electrocardiogram (ECG), stimulation signal (STIM). (**b**) Block diagram of the proportional-integral controller as used during in-silico development. Reference heart rate, HR_ref_, measured heart rate, HR(t), integral gain, K_i_, proportional gain, K_p_, calculated error, e(t), current amplitude, C(t).

Based on the in-silico model, a closed-loop control strategy was developed which uses classical continuous stimulation and cardiac-synchronized stimulation as previously described by Ojeda et al*.*^35^ and Schwartz et al*.*^36^. In cardiac-synchronized stimulation, bursts of stimuli are applied with respect to the cardiac cycle (Fig. 1a). For both stimulation types, charge-balanced cathodic-anodic pulses were used. Continuous stimulation is defined by three main parameters, including the current amplitude (C), pulse width (PW), and frequency (F). In the case of cardiac-synchronized stimulation, there are two more parameters, namely the number of pulses per burst (NP) and the delay between the trigger event and stimulation burst onset (D). The onset of isovolumic ventricular contraction was used as a trigger event for the synchronization.

A sensitivity analysis of the main stimulation parameters to select the proper control variable was conducted. Sobol’s variance decomposition^37^ was employed to identify the influence of the stimulation parameters on the provoked heart rate reduction in the case of open-loop VNS.

A proportional-integral (PI) controller was used that minimizes the error between a desired set heart rate and the measured instantaneous heart rate by adjustment of the stimulation amplitude (Fig. 1b). The stimulation amplitude was limited to 6 mA, since from our experience of previous in-vivo experiments, at this level, side effects like contraction of neck muscles started to occur. The remaining stimulation parameters were fixed (PW = 500 µs, F = 30 Hz, NP = 8, D = 0 ms).

### Quantification of control strategy performance

Similarly to the work of Ugalde et al*.*^28^, the control strategy performance was quantified concerning the rise time (T_r_), the percentual overshoot (%OS), and the steady-state error (SSE). The rise time was defined as the time that is required for the heart rate to go from baseline to 90% of the final value. The percentual overshoot was expressed as a percentage of the steady-state value, and the steady-state error was defined as the mean squared error calculated from the beat in which the system arrived at the steady state. Based on the three performance indicators a cost function was defined as the arithmetic mean of each performance indicator normalized to its respective worst-case requirement:

$$\text{F} = \frac{1}{{3}}\left(\frac{\text{SSE}}{\widehat{\text{SSE}}}\text{+}\frac{\text{\%OS}}{\widehat{\text{\%OS}}}\text{+}\frac{{\text{T}}_{\text{r}}}{\widehat{{\text{T}}_{\text{r}}}}\right)$$

The limits for T_r_, %OS, and SSE were defined as $\widehat{{\text{T}}_{\text{r}}}\text{=}$ 15 s, $\widehat{\text{\%OS}}$=10% and $\widehat{\text{SSE}}$=10 bpm, respectively. Finally, to find the controller gains leading to the best performance in terms of T_r_, SSE, and %OS, the cost function was minimized using Bayesian optimization with 100 evaluations of the cost function starting at the initial values K_p,0_ = 0.1 and K_i,0_ = 0.1.

### In-silico control strategy evaluation and optimization

To generate the virtual study population, n = 50 unique value combinations for relevant model parameters were generated. The parameters were selected based on a previously conducted sensitivity analysis of the vagal cardiac control pathways^32^. Additional model parameters that may potentially exhibit inter-individual variations leading to changes in the cardiac response to VNS were included. The additional model parameters are associated with the sympathetic cardiac control pathway and the cardiovascular system. Eventually, this led to a total of 19 model parameters. A summary of the selected model parameters can be found in Table 1.

**Table 1:** Summary of the computational model parameters that were varied to generate the virtual population.

Parameter	Description	Range	Unit
G_aTv_	Arterial baroreflex gain	0.022–0.037	mmHg^−1^
G_pTv_	Pulmonary stretch reflex gain	0.27–0.45	L^−1^
D_C,symp_	Time delay of sympathetic chronotropic control	2.25–3.75	s
τ_C,symp_	Time constant of sympathetic chronotropic control	1.35–2.25	s
R_as,0_	Systemic vascular resistance	0.73–1.21	mmHg⋅min⋅mL^−1^
µ_fdia_	Mean vagus nerve fiber diameter	1.17–1.43	µm
σ_fdia_	Std. of vagus nerve fiber diameter	0.08–0.1	µm
µ_eldis_	Mean distance from stimulation electrode to vagus nerve fiber	4.4–5.4	mm
σ_eldis_	Std. of distance from stimulation electrode to vagus nerve fiber	0.22–0.28	mm
G_c,vns_	Gain factor of [Ach]_stim_	4.5–7.5	–
k_1_	Rate constant of acetylcholine release into cardiac-vagal synaptic cleft	0.012–0.019	s^−1^
k_H_	Rate constant of acetylcholine hydrolysis in cardiac-vagal synaptic cleft	0.23–0.39	s^−1^
D_D,vns_	Time delay of vagal dromotropic control	0.375–0.625	s
G_D,vns_	Gain of vagal dromotropic control	144.4–240.6	–
PR_0_	Baseline atrioventricular conduction time	0.065–0.12	s
D_E,max_	Time delay of inotropic control pathway	1.5–2.5	s
τ_E,max_	Time constant of inotropic control pathway	0.9–1.5	s
G_E,max_	Inotropic effector gain	0.375–0.625	–
P*_lv_	Parameter influencing end-systolic pressure–volume relationship to adjust left ventricular contractility	202.5–337.5	mmHg

The parameter values were varied in a range of ± 25% which ensured that they remained in a physiologically meaningful range. To ensure proper coverage of the parameter space, Latin Hypercube Sampling (LHS) was employed. Thereby, n = 50 unique points were generated in a 19-dimensional unit hypercube which was then scaled using the previously defined limits of the respective model parameters. The value ranges used for each model parameter can be found in Table 1.

To better understand the control strategy performance in the presence of variations in the relevant model parameters, the influence of the controller gains on the previously defined performance indicators was investigated in the whole virtual study population. Therefore, different value combinations for the controller gains K_p_ and K_i_ in a pre-defined range were tested. Hence, a total of n = 5000 different value combinations for parameter space (K_p_, K_i_) were generated, again using LHS to ensure proper parameter space exploration. The ranges for K_p_ and K_i_ were assigned based on the optimal controller gains initially found in the in-silico model of the single individual. The value ranges have been chosen in a trade-off between the size of the parameter space and sufficient coverage using the 5000 combinations. The ranges that have been used for the generation are K_p_ = [2·10^–5^, 0.01], K_i_ = [2·10^–5^, 0.01] and K_p_ = [2·10^–5^, 0.07], K_i_ = [2·10^–5^, 0.07] for the continuous and the cardiac-synchronized control strategies, respectively.

Then, for every individual in the virtual study population and each of the controller gain combinations a step response test was conducted (set-value for the heart rate reduced from the respective baseline by 20 bpm). To quantify the performance of the control strategy, from each of the simulated step-responses in every individual, three performance indicators (T_r_, %OS, SSE) and a cost function (F) were calculated for every point in the controller gain parameter space and both control strategies.

Using these data, contour plots were generated, illustrating the average performance of the control strategy on the whole study population. From these plots, controller gains were identified that are potentially superior to the ones initially found based on the single virtual individual.

All simulations were performed in MathWorks SIMULINK 9.1 (R2019a).

### Ex-vivo control strategy evaluation

Finally, the control strategy was evaluated in ex-vivo isolated rabbit hearts (n = 6) with intact cardiac vagal innervation testing both sets of controller gains identified in-silico. All experiments were approved by the Institutional Animal Care and Use Committee of the city of Vienna (BMBWF 2020-0.016.858-GZ 2020-0.016.858) and conducted following relevant guidelines and regulations. The experiments were performed and reported in accordance with the ARRIVE guidelines. All surgical procedures were carried out under deep anesthesia (sevoflurane 4%) in ventilated animals.

### Surgical procedure and isolated heart experiment setup

In brief, the right vagus nerve has been dissected starting from the nodose ganglion and moving down to the heart taking special care of cardiac branches given off by the vagus nerve^44^. The innervated-heart preparation was mounted on an erythrocyte-perfused isolated heart system^45^. The heart was perfused under constant pressure (80 mmHg) in Langendorff mode. During the experiment, the vagus nerve was immersed in isotonic sodium chloride to maintain the nerve vital and excitable.

**Figure 2:**
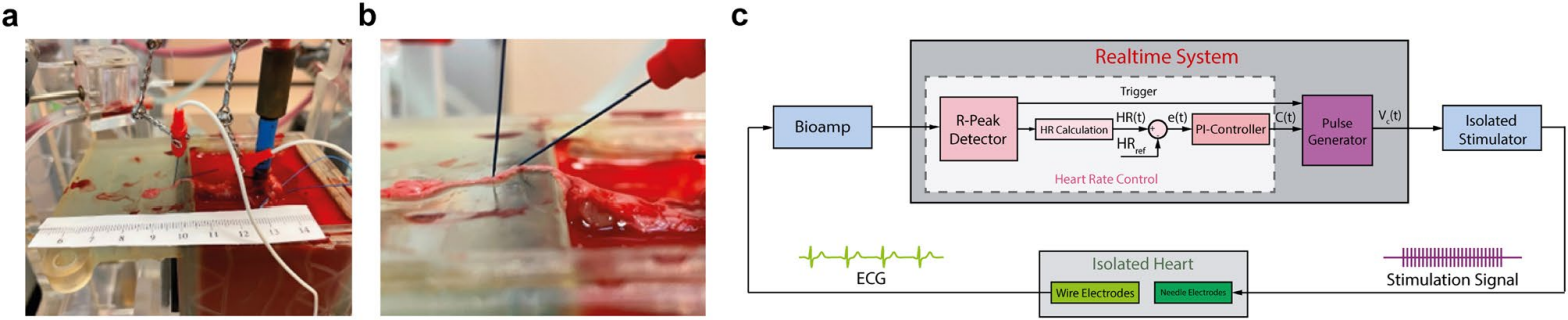
Overview of the experimental setup. (**a**) Isolated heart preparation with needle electrodes inserted into the vagus nerve for stimulation and wire electrodes inserted into the base of the right atrium to measure the electrocardiogram. (**b**) Zoomed-in image of the needle electrodes inserted into the vagus nerve next to the superior cardiac branch with the cathode caudal and the anode cephalad. (**c**) Schematic overview of the hardware-in-the-loop implementation of the control strategy. Reference heart rate, HR_ref_, measured heart rate, HR(t), calculated error, e(t), current amplitude, C(t), control voltage for the isolated stimulator V_c_(t).

Two wire electrodes (MyoStim® Bipolar Bifurcated) were implanted into the right atrium (Fig. 2a) to acquire the electrocardiogram (ECG) which was high-pass filtered at 1 Hz, low-pass filtered at 1000 Hz, and pre-amplified with a gain of 1000 using a differential amplifier (Warner DP-304A).

Two needle electrodes were inserted into the vagus nerve with the cathode and anode approximately 2 mm and 5 mm cephalad to the superior cardiac vagal branch, respectively (Fig. 2b). The needle electrodes were connected to a linear isolated stimulator (Biopac® STMISOLA) which was operated in current-control mode.

### Control strategy evaluation experiment

The control algorithm was implemented using a rapid prototyping system (DSPACE MicroLabBox) whose input and output were connected to the bioelectrical amplifier and the stimulator, respectively, to close the loop (Fig. 2c). For the synchronization of the stimulation with the cardiac cycle, the R-peaks of the ECG were used as the trigger event. Therefore, the ECG was rectified, and the signal maxima were calculated for windows of 5 s. The threshold for R-peak detection was calculated as 75% of the magnitude of the peaks identified from the preceding five windows.

Heart rate reduction steps of 20 bpm from baseline were performed analogously to the in-silico experiments. The stimulation was on for at least 30 s and the stimulation intervals were separated by at least 30 s or until the heart rate returned to baseline. The baseline heart rate was determined from the first 5 min after the heart was mounted to the isolated heart system and Langendorff perfusion had been started.

All data were post-processed calculating the previously described performance indicators.

### Statistical analysis

First, all step responses obtained from the isolated heart experiments were visually inspected, and records exhibiting potential issues such as major arrhythmic events, loosening of electrodes, or changes in stimulability due to nerve degeneration were excluded from the analysis. For all remaining step responses, the respective performance indicators were calculated as previously described and stored in a database for further statistical analysis. Mean and standard deviations were calculated for all performance indicators in the virtual population and the ex-vivo experiments. The performance indicators for initial and optimized controller gains were tested for differences using a two-tailed Student-t test at a significance level of 0.05. All data analysis was performed using Mathworks MATLAB (R2019a).

## Results

### Control strategy development

For the control strategy development, it was vital to select the proper stimulation parameter. Therefore, the influence of main VNS parameters on the provoked heart rate response was investigated in a sensitivity analysis of the in-silico cardiovascular system model. The main effects show that the current amplitude has the greatest influence on the heart rate response, explaining 51.8 ± 1% of the observed variability. It is followed by the pulse width and number of pulses explaining 23.2 ± 0.7% and 8.3 ± 0.2% of the variability in the heart rate responses, respectively. The stimulation frequency and -delay play only a negligible role, explaining only 0.4 ± 0.01% and 0.1 ± 0.01% in the observed variability. Based on these results, the current amplitude has been chosen as the control variable.

### Control strategy evaluation and optimization in the virtual study population

**Figure 3:**
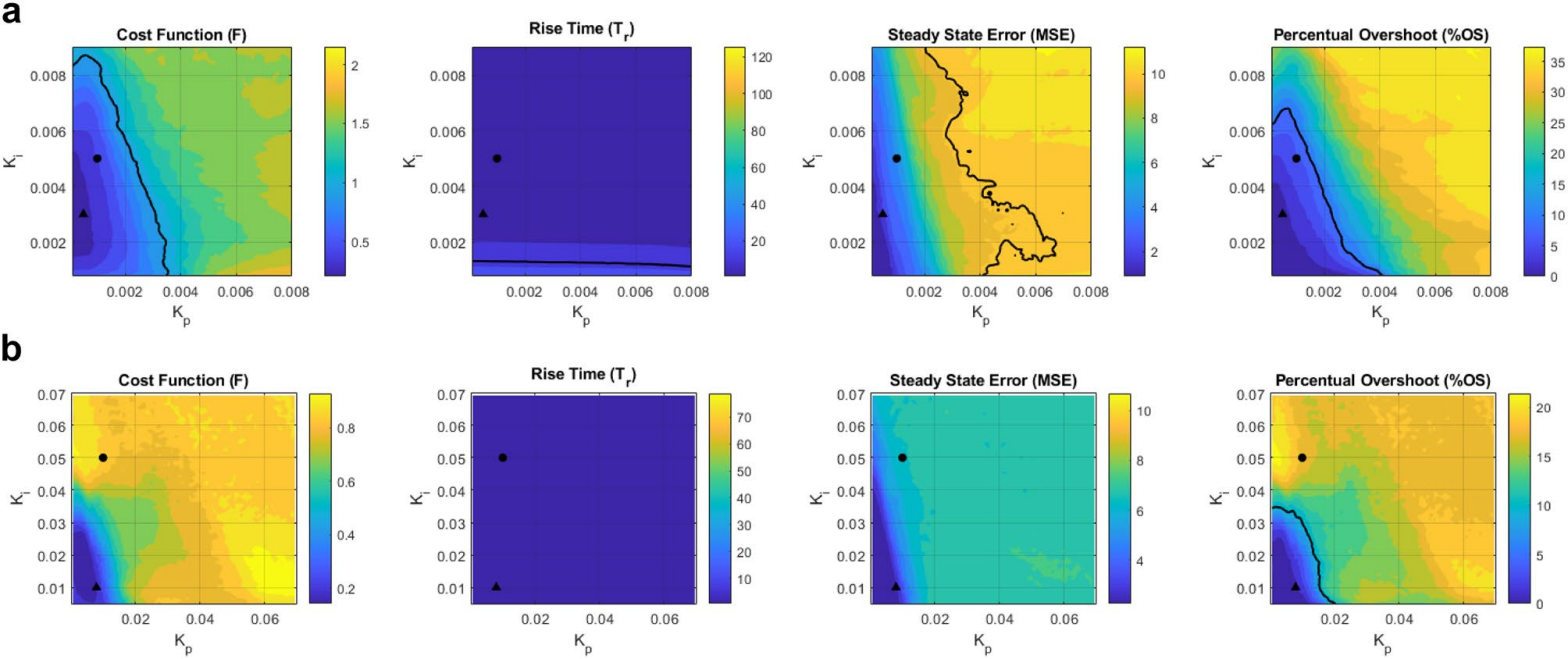
Contour plots of average performance indicators for the whole virtual study population in parameter space (K_p_, K_i_) for (**a**) continuous stimulation and (**b**) cardiac-synchronized stimulation. Initial gain combinations found based on single individual simulation are depicted as point marker and optimized gain combination selected on virtual population are depicted as triangle marker. The acceptable limits for all performance indicators are highlighted by the black contour lines. In the panels where no black contour line is visible, the entire parameters space is part of the optimal domain.

The previously developed controls strategy has been evaluated with respect to three performance indicators, rise time (T_r_), steady-state error (SSE), and percentual overshoot (%OS). The overall controller performance was quantified by a cost function (F), calculated as the mean value of T_r_, SSE, and %OS normalized to their respective maximum acceptable values. Figure 3 shows the contour plots for the average model performance indicators for the whole virtual population. The acceptable limits for the performance indicators are highlighted by the black contour lines. In Fig. 3, we can see a domain of controller gains in the parameter space that all satisfy these limits. In the case of continuous stimulation (Fig. 3a), the initially found controller gains all satisfy the constraints. For the cardiac-synchronized stimulation, the %OS is violated (Fig. 3b).

For the cardiac-synchronized stimulation, the best performing values for K_p_ and K_i_ that were found based on a single virtual individual were 0.01 and 0.05, respectively which led to T_r_ = 5.1 s, SSE = 3.6 bpm, and %OS = 0.1%. These gains were tested on the entire virtual population, for which the performance indicators were T_r_ = 1.3 ± 1.7 s, SSE = 7.3 ± 3.4 bpm, and %OS = 20.5 ± 12.9%.

Based on the results of the analysis of the cardiac-synchronized control strategy performance on the whole virtual study population, an arbitrary gain combination was chosen from the identified optimal domain that potentially performs better than the initially found combination. The selected gains were K_p_ = 0.008 and K_i_ = 0.01 which, in the virtual population, resulted in T_r_ = 9.7 ± 12.4 s, SSE = 4.4 ± 1.5 bpm and %OS = 0.2 ± 0.5%.

For the continuous stimulation, the best performing values for K_p_ and K_i_ that were initially found based on a single virtual individual were 0.001 and 0.005, respectively, resulting in T_r_ = 4.7 s, SSE = 2.8 bpm, and %OS = 8.2%. For the test on the virtual population, these gains led to a mean T_r_ = 3.9 ± 0.4 s, SSE = 4.6 ± 3.4 bpm, and %OS = 7 ± 5.4%.

Also, for the continuous control strategy, gain values were chosen from the identified optimal domain that should lead to improvements in the performance indicators compared to the initially found controller gains. The selected gains were K_p_ = 0.0005 and K_i_ = 0.003 which led to a mean T_r_ = 6.4 ± 0.7 s, SSE = 3.5 ± 3 bpm, and %OS = 3.8 ± 4.2%.

### Ex-vivo control strategy evaluation

**Figure 4:**
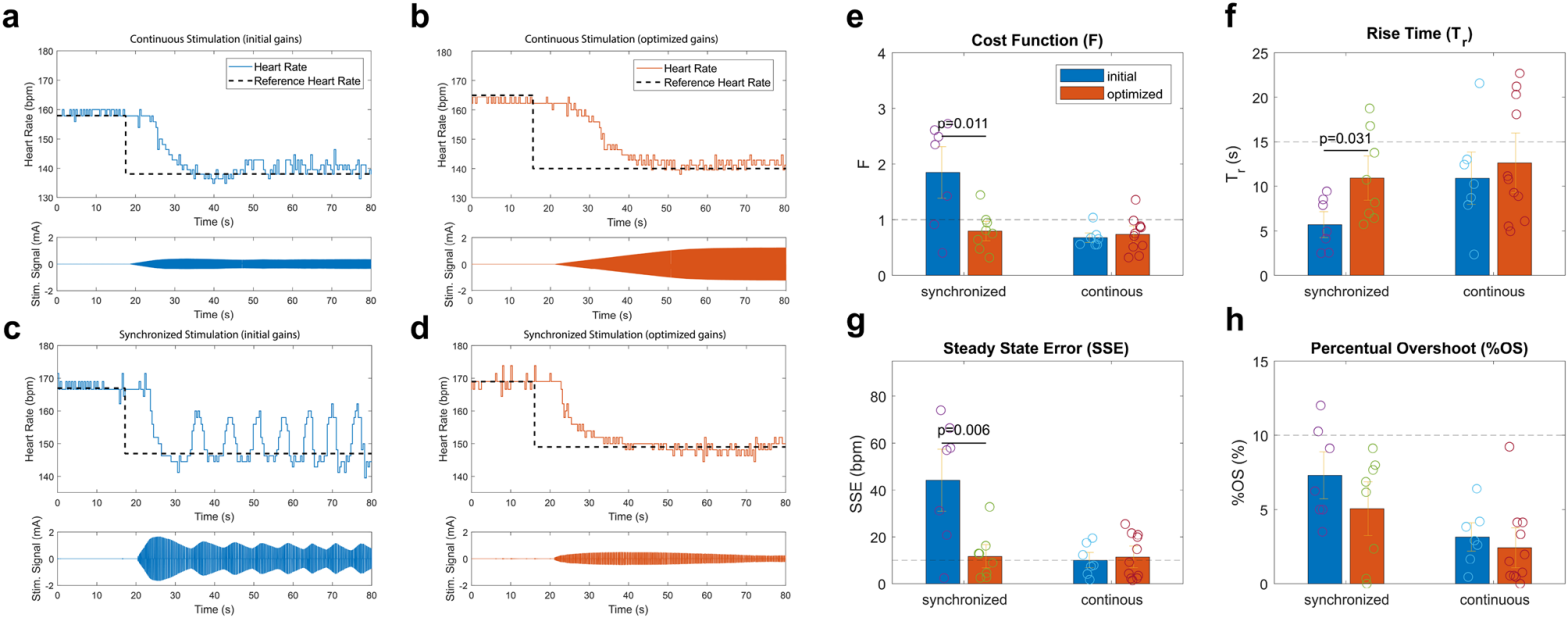
Exemplary heart rate response along with the corresponding stimulation signals for a step reduction of 20 bpm from baseline for (**a**) K_p_ = 0.01, K_i_ = 0.05 and (**b**) K_p_ = 0.008, K_i_ = 0.01 using the continuous control strategy, and for (**c**) K_p_ = 0.001, Ki = 0.005 and (**d**) K_p_ = 0.0005, K_i_ = 0.003 using the cardiac-synchronized control strategy recorded in one isolated heart. Overview of the strategy performance on the whole study population as quantified by (**e**) the cost function, (**f**) rise time, (**g**) steady-state error, and (**h**) percentual overshoot for the initial and optimized controller gains. Differences in the mean performance indicators were tested using a two-tailed Student-t test.

Finally, the control strategy performance was evaluated for the continuous and cardiac-synchronized stimulation and both respective sets of controller gains in isolated Langedorff-perfused rabbit hearts (n = 6) with intact vagal innervation. Figure 4 shows exemplary step responses obtained in one ex-vivo experiment for the initially found gain combinations and the gains optimized based on the virtual population for continuous stimulation (Fig. 4a,b) and cardiac-synchronized stimulation (Fig. 4c,d).

For the cardiac-synchronized stimulation, the optimized controller gains led to a major improvement in the steady-state error at the cost of an increased T_r_. In the case of continuous stimulation, however, one can see that the initially found gains already perform very well and that the optimized gains did not lead to a major improvement in control strategy performance. Qualitatively, this behavior was consistent across all ex-vivo experiments which are quantitatively analyzed in the following.

The control strategy performance was quantified in terms of T_r_, SSE, and %OS which were calculated for the step responses in all ex-vivo experiments. In every experiment, on average, three step responses were performed. During offline analysis, step responses exhibiting potential issues such as major arrhythmic events or substantial reduction in nerve stimulability were removed. In total, 13 records were excluded, resulting in 15 step responses that were used for the analysis below.

For cardiac-synchronized control, in the ex-vivo setting, the initially found gain combination (K_p_ = 0.01, K_i_ = 0.05) led to T_r_ = 4.9 ± 2.9 s, SSE = 44.2 ± 26.5 bpm and %OS = 7.3 ± 3.2% (Fig. 4f–h). Overall, the substantially increased SSE resulted in the exceedance of the limit of the predefined cost function. The optimized gain combination (K_p_ = 0.008, K_i_ = 0.01) resulted in T_r_ = 10.7 ± 4.5 s, SSE = 12.7 ± 9.9 bpm and %OS = 5.1 ± 3.6%, leading to a cost function value well within the predefined limit (Fig. 4e–h). Overall, the optimization led to a substantial improvement of the control strategy performance, leading to a significant reduction in the cost function by 71.3% (Fig. 4e, two-tailed t-test, *p* = 0.011). This reduction was mainly achieved by a vast decrease of the SSE by 31.5 bpm (Fig. 4g, two-tailed t-test, *p* = 0.006). The reduction in SSE was achieved at the cost of T_r_ which was concurrently increased by about 5 s (Fig. 4f, two-tailed t-test, p = 0.031). There was also a slight decrease in %OS by 2.2% which, however, was not statistically significant (Fig. 4h, two-tailed t-test, *p* > 0.05).

For the continuous control, the initially found gain combination (K_p_ = 0.001, K_i_ = 0.005) resulted in T_r_ = 10.2 ± 5.6 s, SSE = 10 ± 6.7 bpm and %OS = 3.2 ± 1.9% and F = 0.6 ± 0.2 (Fig. 4e–h). Hence, with this parameterization, all performance indicator constraints were satisfied. The optimized gain combination (K_p_ = 0.0005, K_i_ = 0.003) resulted in a very similar but slightly worse performance compared to the initial gain set. With these controller gains the performance indicators were T_r_ = 12.5 ± 6.7 s, SSE = 12.6 ± 9.3 bpm and %OS = 2.4 ± 2.7% and F = 0.7 ± 0.3, which, despite the SSE, all are also well within the desired limits (Fig. 4e–h). A two-tailed Student t-test was performed which showed there were no statistically significant differences between performance indicators for the initial and optimized controller gains at a significance level of 0.05.

## Discussion

Persistent sinus tachycardia leads to impaired patient quality of life, possible secondary disease, and a substantially increased risk of cardiac death. It is typically treated by prescription of beta blockers^4^ or antiarrhythmic drugs^3,5^. However, pharmacological treatment is not only associated with various side effects, but also it is often not even possible due to counterindications. Since VNS is known to have a pronounced bradycardic effect, it has the potential as a non-pharmacological treatment alternative to offer patients that are not eligible for conventional therapy. Other non-pharmacological approaches that are already used in clinics include the Medtronic AT500 pacemaker for anti-tachycardia pacing to treat atrial and ventricular tachycardias^48^. Although based on a different effect mechanism this highlights the clinical need for treatment alternatives to traditional pharmacological intervention. In comparison to anti-tachycardia pacing, VNS can be considered a more physiological way to reduce the heart rate since it unfolds its effects through increased parasympathetic efferent cardiac outflow which may also evoke additional cardioprotective effects^49^.

For the application of VNS to manage persistent tachycardia, it is crucial to elicit a reasonable bradycardic response. An inappropriate decrease of the heart rate that does not correspond to an individual’s physiological needs for a given level of physical activity might potentially lead to negative effects like an insufficient blood supply of peripheral tissues resulting in hypoxia. Recent studies of VNS therapy for cardiovascular diseases like atrial fibrillation or hypertension often apply “low-level” vagus nerve stimulation with stimulation intensities below the threshold at which bradycardic effects start to occur^46,47^. In both cases, a bradycardic response is considered an undesired side-effect that should be mitigated if possible.

The goal of the present study was to reduce the heart rate in persistent tachycardia, for which it is explicitly desirable and necessary to apply VNS at an intensity that elicits a substantial bradycardic effect. To lower the heart rate and accurately maintain it at the appropriate level using VNS, it is necessary to employ closed-loop control concepts. The appropriate set heart rate that is attained by the controller may be defined by a physician based on a patient’s physiological condition. In contrast, although open-loop stimulation is often used for neuromodulation therapies, especially for the treatment of neurological disorders, it does not permit precise continuous control of a physiological parameter like the heart rate. This is particularly important to avoid possible negative physiological implications of a bradycardic heart rate that does not match the physiological activity. Moreover, changes in nerve fiber activation thresholds, e.g., from connective tissue formation at the electrode-nerve interface, may lead to inconsistent heart rate responses for given stimulation intensities. Also, the required intensity to elicit a desired heart rate reduction will vary depending on the level of concurrent sympathetic activity and circulating catecholamines. Hence, the application of open-loop VNS for the treatment of persistent tachycardia requires continuous titration through a physician in very short time intervals. Otherwise, there is the risk of either under- or overstimulation resulting in low therapeutic efficacy or adverse events. This titration therefore becomes a process that will not always be feasible in practice. In our opinion, these circumstances justify the preference for closed-loop- over open-loop stimulation for the treatment of persistent sinus tachycardia.

In this work, we developed two control strategies based on an in-silico model, which we validated in the ex-vivo isolated rabbit heart with intact vagal innervation. Since this setup eliminates the chronotropic influence of the autonomic nervous system, it allowed us to quantify the pure performance of the controller.

To select the proper control variable, an in-silico sensitivity analysis of the main VNS parameters has been performed. The sensitivity analysis showed that the current amplitude has the greatest influence on the provoked chronotropic effect, followed by pulse width, number of pulses, frequency, and delay in this order. These results are consistent with the findings of Ojeda et al*.*^35^. In their study, a similar sensitivity analysis was performed based on data collected from VNS experiments in anesthetized sheep showing the same order of importance of stimulation parameters with quantitatively similar results for Sobol’s first-order indices. Because of its preeminent influence on the provoked heart rate response, we selected the current amplitude as the control variable. However, depending on the employed implantable stimulator, pulse width might pose a better target concerning energy efficiency.

For the evaluation of the in-silico developed and optimized closed-loop VNS control strategy, a novel approach was pursued by using ex-vivo isolated rabbit hearts with intact vagal innervation^38^. This allowed evaluation of the controller performance in the complete absence of possible confounding mechanisms such as anesthetic effects or physiological feedback loops like the arterial baroreflex. Although Ng et al*.* have previously used the in-situ isolated rabbit heart with intact vagal innervation to study the acute cardiac effects of vagus nerve stimulation^39–43^, to the best of our knowledge, that is the first study that uses this model for the investigation of closed-loop VNS strategies for heart rate control in a novel fully ex-vivo setting.

In the past decades, acute chronotropic effects of open-loop VNS have been extensively studied in anesthetized animals^50–53^ and in-situ isolated rabbit hearts^39–43^. Previous investigators have also evaluated closed-loop VNS for heart rate control in acute in-vivo experiments in anesthetized animals^18,27–29,54–57^ The disadvantage of the in-vivo model is that anesthetic effects and physiological variations cannot be excluded, which may potentially affect the assessment of the control strategy performance^28^.

Previous studies demonstrated the cervical vagus nerve as a stimulation target for closed-loop heart rate control^18,27–29,54–57^. Since the cervical vagus nerve contains afferent and efferent nerve fibers innervating virtually any organ, stimulation at this level is potentially accompanied by side effects like contraction of neck muscles, hoarseness, or hiccups^10^. By stimulating proximal to the superior cardiac branch, we may improve the selectivity of the stimulation maximizing the chronotropic response while reducing side effects associated with stimulation at the cervical level. This novelty represents a major improvement to standard cervical VNS, though, at the cost of increased invasiveness of the implantation procedure. However, this drawback might not be relevant with the steady advance in minimally invasive techniques.

For cardiac-synchronized stimulation, the analysis conducted on the virtual study population shows that the controller gains that were initially found based on the in-silico model of the single individual were part of the optimal domain of controller gains leading to satisfaction of the predefined limit for the cost function. According to the results of the virtual study population, however, there was the possibility to further increase the control strategy performance by using the optimized controller gains. Based on the in-silico predictions, this reduction in controller gains could lead to a 62% improvement in the cost function by a concurrent reduction in SSE and %OS at the cost of an increase in T_r_. In the ex-vivo experiment, the optimized controller gains indeed led to a vast decrease in the cost function of 56% which was even greater than predicted in the in-silico experiment. This decrease in the cost function could be mainly achieved by a strong reduction of about 71% in the SSE. There was also a slight concurrent decrease of about 30% in the %OS and an increase in the T_r_ of about 47%. Overall, the in-silico optimized control strategy led to very good overall performance in terms of T_r_, SSE, and %OS. Moreover, the test in the virtual study population suggests satisfying performance in a broad variety of patients exhibiting variations in physiological parameters associated with the cardiovascular system and its autonomic control.

In the case of continuous stimulation, the in-silico analysis in the virtual population shows already excellent controller performance. However, the simulations suggest, that by reducing the K_p_ and K_i_, the %OS could be further increased. Looking at the results from the ex-vivo experiments, the %OS could indeed be slightly improved by 30% by using the optimized controller gains. However, the overall control strategy performance quantified by the cost function could not be improved. Indeed, there was even a slight degradation in the control strategy performance when using the optimized controller gains compared to the initially found gains. None of the differences in the performance indicators between initial- and optimized gains were statistically significant.

Although the control strategy performance was improved in the ex-vivo experiment in a similar way as suggested by the in-silico model, there is a general discrepancy between our experimental- and simulation results. Especially for the cardiac-synchronized stimulation, the ex-vivo experiments showed substantially higher SSE than predicted by the computer model. A possible reason is that the employed single-cell model of the sinoatrial node might not be able to properly capture the phase sensitivity to vagus nerve stimulation observed experimentally. Therefore, adjustment of the stimulation delay might influence the performance of the cardiac-synchronized control strategy and should be investigated in future experiments.

For the proposed control strategies there is the potential risk of reducing the heart rate to a level that does not match the individual’s physiological needs for the given state of physical activity. However, through apt adjustment of the desired set heart rate, this issue can be properly addressed. Since persistent sinus tachycardia is primarily relevant for the resting condition, a physician can define an appropriate resting heart rate corresponding to the patient’s specific condition. For handling the transition to an exercise condition, a mechanism of VNS withdrawal similar to rate-responsive pacing can be envisioned. Here sensor data, e.g., from wearable accelerometers can be used to estimate the level of physical activity and to provide a proper set heart rate to the closed-loop controller that matches the patient’s physiological needs.

The method that was used for the online detection of R-peaks in the ECG is a very simple one, that was demonstrated to be sufficient for the very well-controllable ex-vivo experimental condition. Nevertheless, this technique might not be able to reliably detect R-peaks with sufficient accuracy in the in-vivo condition, e.g., due to potential misdetections caused by changes in signal amplitude. Therefore, for future work, especially in the case of long-term in-vivo studies, the implementation of more sophisticated algorithms for R-peak detection, like the standard algorithm of Pan and Tompkins^58^ is planned.

Recent work is directed toward the evaluation of the control strategy performance in the isolated working heart setup in which the circulation is closed, and physiological pressure conditions are produced in the heart chambers. This will allow us to analyze the concurrent effects of the closed-loop vagus nerve stimulation on atrioventricular conduction time and ventricular contractility. Future work will be dedicated to the incorporation of algorithms for estimation of physical activity to achieve rate responsive-pacing and hence ensure heart rates that fit the hemodynamics needs for a given level of physical activity.

To conclude, we applied an in-silico model of the acute cardiac effects of cervical vagus nerve stimulation for the development of closed-loop heart rate control strategies. The control strategy performance was assessed and optimized based on a virtual study population in which relevant model parameters were varied to resemble expected physiological interindividual variations. Finally, the control strategy was evaluated in Langendorff-perfused rabbit hearts with intact cardiac innervation. The in-silico-developed control strategy resulted in overall good and reproducible performance, satisfying all constraints for controller rise time, percentual overshoot, and steady-state error.

## Data Availability

The data that support the findings of this study are available from the corresponding author upon reasonable request.
